# Safety and Efficacy of Micronized Acellular Dermal Matrix Injection for Correction of Moderate to Severe Nasolabial Folds: A Double-Blind, Multicenter, Randomized Controlled, Non-inferior Clinical Trial

**DOI:** 10.1007/s00266-025-05494-4

**Published:** 2025-12-11

**Authors:** Chen Zhang, Xu Chang, Liyang Chen, Hang Wang, Ge Feng, Wei Zheng, Linfeng Li, Wei Xu, Xiangdong Qi, Xuan Jiang, Xiao Long, Nanze Yu, Lu Chen, Nannan Long, Hongsen Bi

**Affiliations:** 1 Department of Plastic Surgery, Peking University Third Hospital, Haidian District, 49 North Garden Road, Beijing, 100191 China; 2 Department of Plastic Surgery, West China Hospital of Stomatology, Sichuan University, 14, Section 3, Renmin South Road, Chengdu, 610041 China; 3 Department of Dermatology, Beijing Friendship Hospital, Capital Medical University, 95 Yongan Li, Xicheng District, Beijing, 100050 China; 4 Department of Plastic Surgery, Zhujiang Hospital of Southern Medical University, 253 Industrial Avenue, Haizhu District, Guangzhou, 510000 China; 5 Department of Plastic and Aesthetic Surgery, Peking Union Medical College Hospital, Chinese Academy of Medical Sciences and Peking Union Medical College, 41, Damucang Hutong, Xicheng District, Beijing, 100032 China; 6 Beijing Ruijian High-Tech Biological Technology Co., Ltd, Room 202, Building 15, 1, Chaoqian Road, Changping District, Beijing, 102200 China

**Keywords:** Micronized acellular dermal matrix (mADM), Nasolabial folds (NLF), Filler injection, Aesthetic rejuvenation, Randomized controlled trial (RCT)

## Abstract

**Background:**

Nasolabial folds (NLFs) are prominent facial lines that deepen with age and may adversely affect facial aesthetics and psychosocial confidence. Injectable fillers, including micronized acellular dermal matrix (mADM), offer minimally invasive correction; however, clinical evidence for mADM’s safety and efficacy remains limited. We conducted a double-blind, multicenter, randomized controlled trial to compare an mADM filler (Regenfil) with a regulatory agency–approved cross-linked collagen filler (Sunmax Collagen Implant I-Plus).

**Methods:**

Adults aged ≥18 years with Wrinkle Severity Rating Scale (WSRS) grade 3 or 4 NLFs were randomized to receive mADM or collagen. Six weeks after the initial treatment, a protocol-defined supplemental injection was permitted if additional correction was needed. Outcomes were assessed 6 weeks, 3 months, and 6 months after the last treatment. Efficacy endpoints included blinded-evaluator WSRS, Global Aesthetic Improvement Scale (GAIS) rated by injectors and participants, and participant satisfaction. The prespecified primary endpoint was the percentage of participants achieving a >1-grade WSRS improvement simultaneously on both left and right NLFs. Safety was evaluated by monitoring device-related adverse events and local injection-site reactions.

**Results:**

Of 202 randomized participants, 175 completed all required follow-ups for safety and efficacy (mADM, n=86; collagen, n=89). The mADM group required lower mean filling volumes and fewer supplemental injections than the collagen group (*P*<0.05). At 3 months after the last treatment, the proportion meeting the primary endpoint was 88.4% with mADM versus 85.4% with collagen (*P*>0.05). At 6 months, efficacy was 70.9% with mADM and 69.7% with collagen (*P*>0.05). Apart from GAIS and participant satisfaction at 6 weeks, which favored collagen, no statistically significant differences were observed between groups across other efficacy endpoints and timepoints. The most common adverse events were hardening, redness, and skin discoloration at injection sites, occurring at similar frequencies in both groups (*P*>0.05). Early local reactions such as swelling and pain were more frequent with mADM (*P*<0.05), but were predominantly mild to moderate and resolved within one week. No treatment-related serious adverse events were reported.

**Conclusion:**

In this multicenter, randomized, double-blind trial, mADM demonstrated safety and effectiveness for correcting moderate-to-severe NLFs, with outcomes comparable to a cross-linked collagen filler through 6 months. Compared with collagen, mADM achieved similar correction with less product volume and fewer supplemental injections, while showing a transient increase in early local reactions that were self-limited. These findings support mADM as a minimally invasive, injectable tissue-regenerative bioscaffold and a viable alternative for facial rejuvenation.

**Bullet Point List:**

Comparable Efficacy: mADM filler demonstrated similar effectiveness to collagen fillers in reducing moderate-to-severe nasolabial folds.

Enhanced tissue remodeling: Histological analysis showed that mADM promotes collagen deposition, fibroblast proliferation, and vascularization.

Minimally Invasive & Safe: While early adverse reactions (swelling, pain) were slightly higher with mADM, they were mild, self-limiting, and resolved within a week.

Lower Injection Volume Needed: mADM required significantly less filler volume and fewer supplemental injections compared to collagen fillers.

**Level of Evidence I:**

This journal requires that authors assign a level of evidence to each article. For a full description of these Evidence-Based Medicine ratings, please refer to the Table of Contents or the online Instructions to Authors www.springer.com/00266.

**Supplementary Information:**

The online version contains supplementary material available at 10.1007/s00266-025-05494-4.

## Introduction

Facial aging is most noticeable in the mid-face. Being the focal point of facial expressions and attention, the cheeks play a key role in defining facial contours. Congenital fat insufficiency and age-related volume loss can significantly impact facial aesthetics [1]. Facial aging in Chinese women is most prominent between the ages of 40 and 49, with facial volume loss being the primary manifestation, such as tear troughs and deepened NLFs [2].

Nasolabial folds (NLFs) are indented facial lines that separate the cheek and upper lip regions, extending from the nasal wings to the corners of the mouth and sometimes below. The depth and shape vary among individuals, deepening with age and serving as a hallmark of facial aging [3].

Various treatments are available for correcting NLFs, including filler injections, surgical procedures, and laser or radiofrequency therapies [4]. Energy-based treatments stimulate skin regeneration through wound healing processes but often have delayed effects and require multiple sessions [5]. Traditional surgical approaches (e.g., endoscopically assisted midface lift and open rhytidectomy) are associated with significant trauma and long recovery time. Filler injection, being minimally invasive, has currently become the primary treatment for improving NLFs [6].

Injectable fillers include synthetic polymers, bioceramics, natural polymers, and tissue-engineered materials. They achieve corrective effects by different mechanisms, filling, stimulating or scaffolding for new tissue formation. Acellular dermal matrix (ADM), due to its low immunogenicity and ability to recellularize and revascularize into the patient’s own new tissue, is a highly promising tissue-engineered material for plastic and aesthetic surgery [7]. Micronized injectable ADM (mADM) could be an ideal subcutaneous filler for the reconstruction of a bio-scaffold microenvironment for facial rejuvenation via tissue regeneration, correcting NLFs and restoring youthful appearances [8]. However, there is little research evidence to support the wide aesthetic use of the mADM filler.

In this clinical trial, we evaluated the safety and efficacy of a new injectable mADM filler for correcting NLF wrinkles and depressions. As the double-blind, multicentre, randomized controlled, non-inferior clinical study, a regulatory agency-approved collagen filler (Collagen Implant I-Plus, Sunmax Biotechnology Co. Ltd. Taipei, China) was used as the control device.

## Methods

### Study Design

The prospective clinical trial strictly adhered to the Declaration of Helsinki and Chinese clinical trial regulations. The prospective clinical trial, registered in the Clinical Trial Registry (ChiCTR2300067749), was designed to enroll participants aged 18 and older with moderate to severe nasolabial folds (i.e., the wrinkle severity rating scale (WSRS) grades of 3 or 4). It was conducted across five research hospitals in China after the ethics approval of the trial was obtained from each of the five participating centers. The exclusion criteria for participants included: (1) Individuals being allergic to porcine-derived materials; (2) Individuals with autoimmune diseases or a history of severe allergies; (3) Individuals with coagulation disorders; (4) Individuals with scars, tattoos, piercings, facial hair, or skin conditions in the injection area or mid-face region, or those who had undergone relevant treatments (e.g., facial fillers, botulinum toxin injections, dental treatments) that might affect the evaluation of the study; (5) Individuals with severe functional impairments of vital organs (e.g., heart, brain, lungs); (6) Individuals with a history of severe malignant tumors within the past five years before enrollment; (7) Pregnant or breastfeeding individuals, or those planning to become pregnant during the study; (8) Any other conditions deemed unsuitable for enrollment by the researchers.

Participants were voluntary, and each of them signed the form of informed consent. The treatment to correct the NLFs was the filler injection by aesthetic doctors. On Day 0, the participant received the first injection for both NLFs with either the mADM filler or the collagen filler. Six weeks after the first treatment, the participants could choose to receive a supplemental (second) injection if the results showed asymmetry between the two sides or the improvement in WSRS score on either side was less than one grade, or if the injector deemed the aesthetic result suboptimal. The efficacy and safety evaluations were conducted at six weeks, three months, and six months after the supplemental injection treatment or after the first injection treatment for those who did not receive supplemental injection. Primary endpoints were the WSRS improvement after three months and six months. Secondary endpoints were the WSRS improvement at six weeks, Global Aesthetic Improvement Scale (GAIS) scores and participant satisfaction at six weeks, three months and six months. Safety evaluation was done by monitoring adverse events.

### Randomization

The study used a centralized randomization scheme, where the use of the mADM filler or the collagen filler was allocated. The randomization was done using the SAS 9.4 program with the 1:1 ratio between the mADM filler and the collagen filler (i.e., the odd of 50% allocation) in the blocks of four subjects.

### Endpoint Assessment

In each research center, aesthetic doctors who were not involved in the treatment of participants and blinded to the treatment allocation, were appointed and trained as the WSRS assessors to perform the on-site evaluations. It was recommended that the same independent blinded assessor conducted the evaluations at each visit to maintain consistency throughout the study period. Prior to treatment, each enrolled participant was assessed by the blinded evaluator for the severity of NLFs using the WSRS (Supplementary Table 1). After treatment, the improvement of NLFs was evaluated by the blinded evaluator at six weeks, three months and six months after the last injection. The improvement of the Global Aesthetic Improvement Scale (GAIS, Supplementary Table 2) was evaluated by both injectors and participants. The satisfaction rating was based on the participant’s satisfaction survey form (Supplementary Table 3).

Safety evaluation was done by monitoring all device- or procedure-related serious adverse events throughout the study. Local reactions at the injection sites were documented by the participants in their research diaries within 30 days after the first and supplemental injection treatment.

### Test Materials

The mADM filler (Regenfil) (Beijing Ruijian High-Tech Biotechnology Co., Ltd. (Beijing, China) was made of decellularized and antigen-eliminated porcine dermis matrix microparticles suspended in saline. The control device was Sunmax Collagen Implant I-Plus (Sunmax Biotechnology Co. Ltd. Taipei, China). This collagen filler has been widely used in China for more than 15 years, with the proofing record of good safety and efficacy.

### Statistical Analysis

The improvement rate was defined as the percentage of participants whose WSRS grades for both NLFs decreased by at least one grade (≥1 point) from baseline, as assessed by blinded evaluators. The non-inferiority margin was specified to be 10% for the primary WSRS improvement. The GAIS improvement was defined as the percentage of participants having a GAIS score of “being very much improved, much improved or somewhat improved”, as evaluated by the injectors and participants. The satisfaction rate is defined as the percentage of participants who were very satisfied or satisfied according to the participant satisfaction survey.

Statistical analyses were conducted using SAS software version 9.4. Quantitative data were expressed as means and standard deviations ($\overline{x}$ ± s). Statistic comparisons for quantitative variables were performed using t-tests for normally distributed data with equal variances or Wilcoxon rank-sum tests for non-normal or unequal variance data. Comparisons for categorical variables were made using chi-square tests or Fisher's exact tests (if chi-square tests were not applicable). Comparisons for ordinal variables were performed using the Wilcoxon rank-sum tests or Cochran-Mantel-Haenszel (CMH) tests. A *p*-value < 0.05 (two-sided) was considered statistically significant.

## Results

**Fig. 1:**
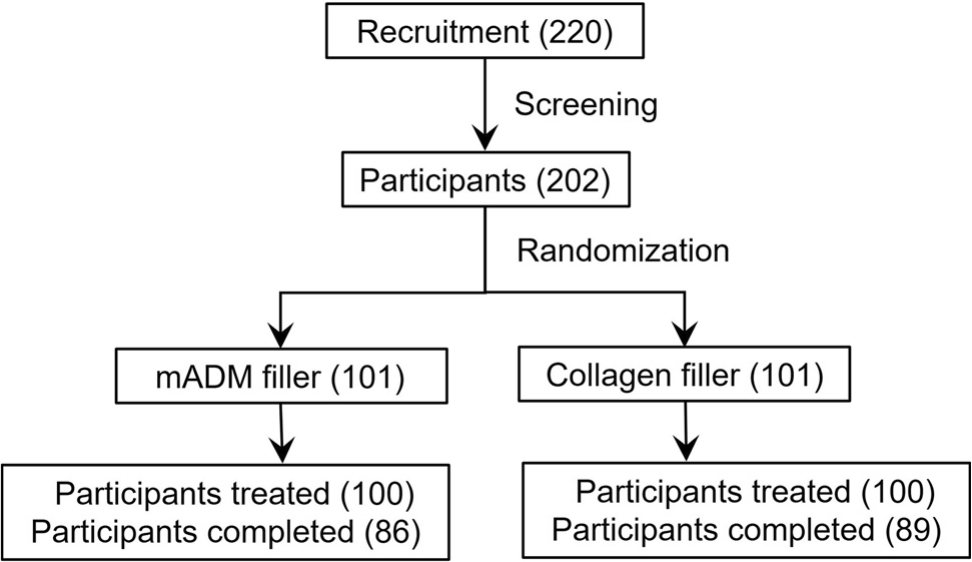
The flowchart of the clinical trial (recruitment, screening, inclusion and exclusion of participants)

In this clinical trial 220 patients were screened and signed the informed consent form in five research hospitals in China. The study enrolled a total of 202 participants and 200 of them received mADM filler or collagen filler treatment. The flow chart of screening, inclusion and exclusion of the participants is presented in Fig. 1, and the enrollment of participants in five hospitals is presented in Supplementary Table 4. There was a total of 27 drop-offs (13.4%) during the study. The mADM filler group included 86 completed participants, 78 female (90.7%) and 8 male (9.3%), whereas the collagen filler group included 89 completed participants, 87 female (97.8%) and 2 male (2.2%).

There was no statistical difference in the baseline characteristics of participants between the mADM filler group and the collagen filler group (*p* > 0.05) (Table 1). Among the 86 completed participants in the mADM filler group, 68 participants (79.1%) had moderate NLF wrinkles and 18 participants (20.9%) severe NLF wrinkles. Among the 89 completed participants in the collagen filler group, 69 participants (77.5%) had moderate NLFs and 20 participants (22.5%) severe NLFs. Therefore, the comparison of WSRS grade assessments prior to treatments was no statistical different between the two groups (*p* > 0.05) (Table 2).

**Table 1:** The baseline characteristics of participants who completed the clinical trial (mean ± SD)

Characteristic	mADM filler group (n=86)	Collagen filler group (n=89)	*p*-Value
Gender (male/female)	8/78	2/87	0.055
Age (years)	44.7±11.2	43.5±10.2	0.414
Height (cm)	162.6±6.7	161.0±5.7	0.165
Weight (kg)	59.2±8.0	58.0±7.9	0.187
BMI (kg/m^2^)	22.4±2.6	22.4±3.2	0.905

**Table 2:** WSRS Grades of participants who completed the clinical trial during screening time

Characteristic	WSRS Grade	mADM filler Group (n=86)	Collagen filler Group (n=89)	*p*-Value
Left NLF	Grade 1 (None)	0 (0.0%)	0 (0.0%)	0.805
	Grade 2 (Mild)	0 (0.0%)	0 (0.0%)	
	Grade 3 (Moderate)	68 (79.1%)	69 (77.5%)	
	Grade 4 (Severe)	18 (20.9%)	20 (22.5%)	
	Grade 5 (Very Severe)	0 (0.0%)	0 (0.0%)	
Right NLF	Grade 1 (None)	0 (0.0%)	0 (0.0%)	0.805
	Grade 2 (Mild)	0 (0.0%)	0 (0.0%)	
	Grade 3 (Moderate)	68 (79.1%)	69 (77.5%)	
	Grade 4 (Severe)	18 (20.9%)	20 (22.5%)	
	Grade 5 (Very Severe)	0 (0.0%)	0 (0.0%)	

In the mADM filler group, the averaged total filling volume of 86 participants was 1.8 ± 0.8 mL for the left NLF and 1.8 ± 0.8 mL the right NLF, with only 60 participants (69.8%) receiving the supplemental injection. In the collagen filler group, the averaged filling volume of 89 participants was 2.1 ± 0.7 mL for the left NLF and 2.1 ± 0.7 mL for the right NLF, with 74 participants (83.2%) having the supplemental injection. There was significantly more supplemental injection and filling volume in the collagen filler group than in the mADM filler group (*p* < 0.05) (Table 3).

**Table 3:** The filling volume of participants who completed the clinical trial and the number of participants with the supplemental injection and (mean ± SD)

Injection time / filling volume	mADM filler group (n = 89)	Collagen filler group (n = 89)	*p*-Value
With supplemental injection	60 (69.8%)	74/89 (83.2%)	0.037
Left NLFs	1.8 ± 0.8 (mL)	2.1± 0.7 (mL)	0.017
Right NLFs	1.8 ± 0.8 (mL)	2.1 ± 0.7 (mL)	0.009

**Fig. 2:**
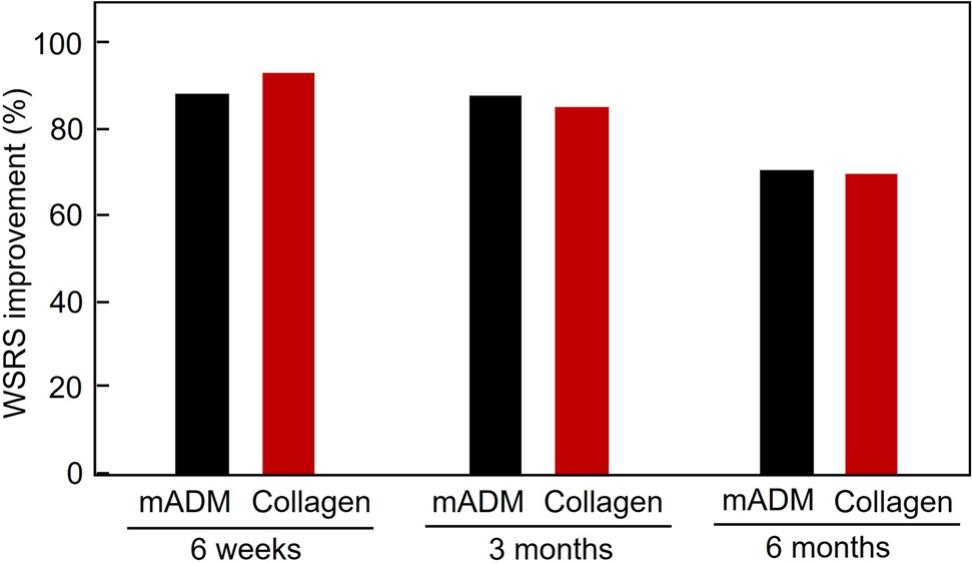
The efficacy of the WSRS improvement after nasolabial fold correction. Participantswere evaluated by blinded aesthetic doctors at 6 weeks, 3 months, and 6 months post-final injection. No statistical difference was observed between the mADM filler and the collagen filler at any time point (*p* > 0.05)

As the primary endpoints, the efficacy for the improvement for WSRS scores (as assessed by blinded evaluators) in the mADM filler group were 88.4% (76/86) and 70.9% (61/86) at three months and six months following the final injection respectively, whereas the efficacy for the improvement in the collagen filler group were 85.4% (76/89) and 69.7% (62/89) at three months and six months following the final injection, respectively (Table 4). The non-inferiority margin of this clinical trial was specified to be 10% for the primary WSRS improvement. The difference between the mADM filler and the collagen filler (mADM - Collagen) was 3.0% (95% CI 6.8–13.4%, *p* < 0.05) at three months, whereas the difference was 1.2% (95% CI 12.3–14.8%, *p* = 0.10) at six months. Therefore, the mADM filler and the collagen filler were substantially equivalent in the efficacy for NLFs correction, and the mADM filler was non-inferior to the collagen filler. The efficacy for the WSRS improvement at the short time 6 weeks was 88.4% and 93.1% in the mADM filler and collagen filler, respectively, with no statistical difference (*p* > 0.05) (Fig. 2).

**Table 4:** The improvement of WSRS grades and efficacy at three months and six months after the final injection

NLF side	WSRS Grade	mADM filler group (n=86)	Collagen filler group (n=89)	*p*-Value
Three months				
Left NLF	Grade 1 (None)	9 (10.5%)	10 (11.2%)	0.948
	Grade 2 (Mild)	57 (66.3%)	56 (62.9%)	
	Grade 3 (Moderate)	18 (20.9%)	22 (24.7%)	
	Grade 4 (Severe)	2 (2.3%)	1 (1.1%)	
	Grade 5 (Very Severe)	0 (0.0%)	0 (0.0%)	
Right NLF	Grade 1 (None)	7 (8.1%)	11 (12.4%)	0.856
	Grade 2 (Mild)	60 (69.8%)	56 (62.9%)	
	Grade 3 (Moderate)	18 (20.9%)	21 (23.6%)	
	Grade 4 (Severe)	1 (1.2%)	1 (1.1%)	
	Grade 5 (Very Severe)	0 (0.0%)	0 (0.0%)	
Efficacy (%)		76/86 (88.4%)	76/89 (85.4%)	0.561
Six months				
Left NLF	Grade 1 (None)	2 (2.3%)	7 (7.9%)	0.404
	Grade 2 (Mild)	50 (58.1%)	44 (49.4%)	
	Grade 3 (Moderate)	28 (32.6%)	37 (41.6%)	
	Grade 4 (Severe)	6 (7.0%)	1 (1.1%)	
	Grade 5 (Very Severe)	0 (0.0%)	0 (0.0%)	
Right NLF	Grade 1 (None)	4 (4.7%)	7 (7.9%)	0.641
	Grade 2 (Mild)	48 (55.8%)	44 (49.4%)	
	Grade 3 (Moderate)	28 (32.6%)	36 (40.5%)	
	Grade 4 (Severe)	6 (7.0%)	2 (2.3%)	
	Grade 5 (Very Severe)	0 (0.0%)	0 (0.0%)	
Efficacy (%)		61/86 (70.9%)	62/89 (69.7%)	0.855

**Fig. 3:**
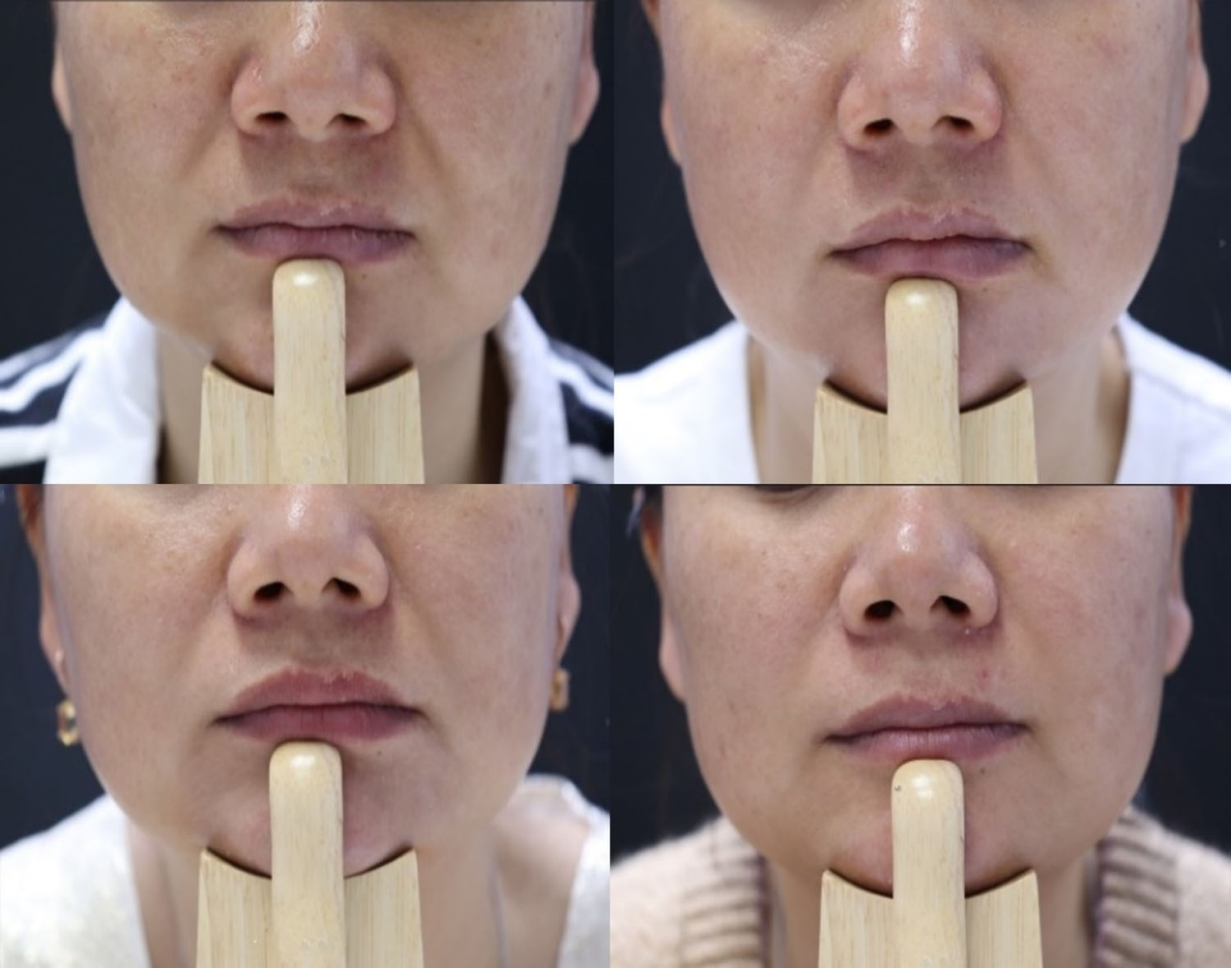
Representative photos showing the NLF correction with the mADM filler for a 40-year old female. Top left: Before injection (WSRS grade 3); top right: Six weeks after the final injection (WSRS grade 2); bottom left: Three months after the final injection (WSRS grade 2); bottom right: Six months after the final injection (WSRS grade 2)

**Fig. 4:**
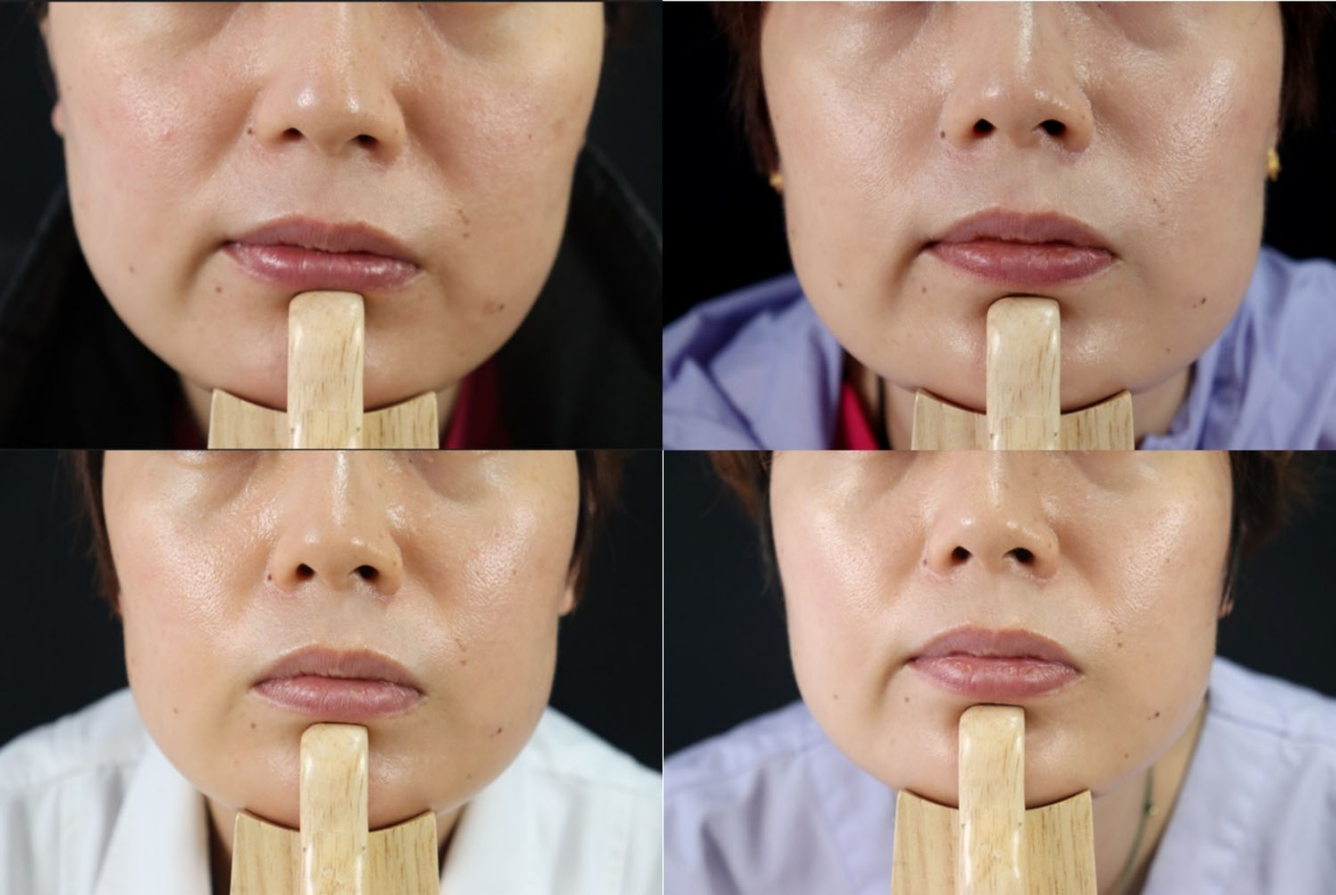
Representative photos showing the NLF correction with the collagen filler for a 41-year old female. Top left: Before injection (WSRS grade 3); top right: Six weeks after the final injection (WSRS grade 2); bottom left: Three months after the final injection (WSRS grade 2); bottom right: Six months after the final injection (WSRS grade 2)

**Fig. 5:**
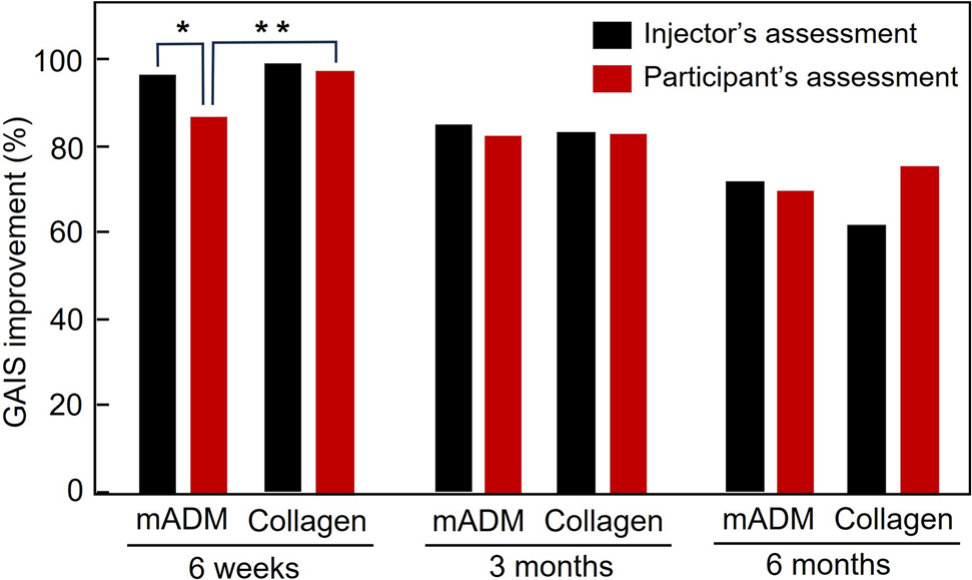
GAIS improvementafter nasolabial fold correction as evaluated by the injector and participants at 6 weeks, 3 months, and 6 months post-final injection. At 6 weeks post-injection, the assessments between injectors and participants differ significantly (*p < 0.05), and participant assessments favored the the collagen filler group (**p < 0.01). At 3 months and 6 months, no significant differences were observed in assessmentss between two fillers and between injectors and participants (*p* > 0.05)

For the GAIS improvement evaluated by injectors, the mADM filler group had an improvement rate of 96.5%, 84.9% and 72.1% at six weeks, three months and six months post-final injection respectively, as compared to 98.9%, 83.2% and 61.8% in the collagen filler group (Figs. 3 and 4), with no statistical difference at any timepoints (*p* > 0.05). For GAIS improvement self-assessed by participants, the mADM filler group had an improvement rate of 87.2%, 82.6% and 69.8% at six weeks, three months and six months post-final injection respectively, as compared to 97.7%, 83.2% and 75.3% in the collagen filler group. The participant assessed more favorable to the collagen filler at the 6-week timepoint (*p* < 0.01), but no difference at two later timepoints (*p* > 0.05) (Fig. 5).

**Fig. 6:**
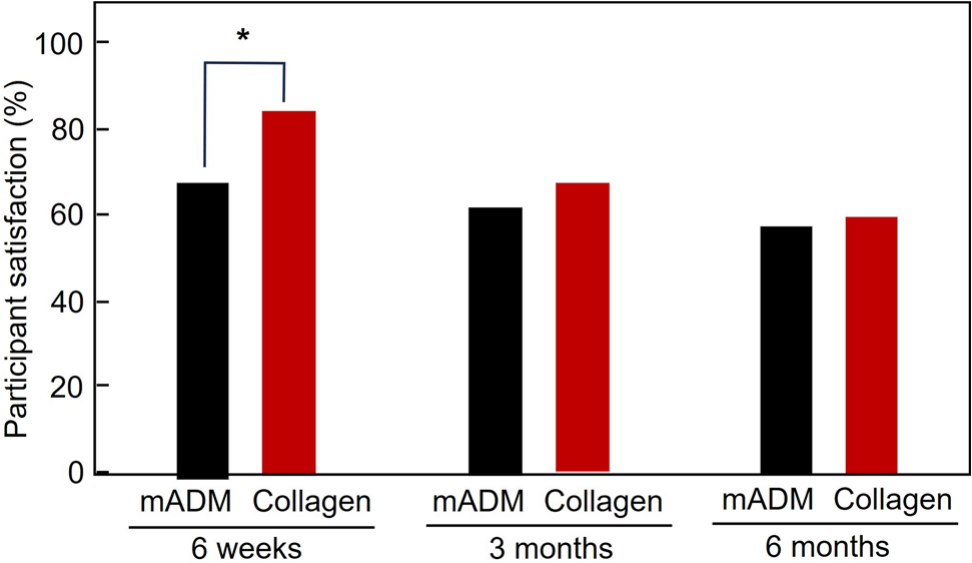
Participant satisfaction after nasolabial fold correction at 6 weeks, 3 months, and 6 months after the final injection. At 6 weeks, the collagen filler showed significantly higher satisfaction rates as compared to the mADM filler (*p < 0.05). No significant differences in satisfaction were observed between two fillers at 3 months and 6 months (*p* > 0.05)

With regard to the participant satisfaction, the mADM filler had a lower satisfaction rate of 67.4% than the collagen filler (83.9%) at six weeks post-final injection, showing a statistically significant difference (*p* < 0.05). The participant satisfaction rates decreased to 61.6% in the mADM filler and 67.4% in the collagen group at three months. The satisfaction rates decreased further to 57.0% in the mADM filler and 59.6% in the collagen group at six months, with no statistically significant difference at two later timepoints (*p* > 0.05) (Fig. 6).

In terms of safety, no severe adverse events related to the devices or injection procedures were reported throughout the study. Common local reactions included pain, swelling, redness, bruising, and hardening. The incidences of pain (mADM filler 40.7% vs collagen filler 10.1%), swelling (mADM 45.4% vs collagen filler 13.5%), and skin temperature increase (mADM filler 8.1% vs collagen filler 1.1%) were all higher in the the mADM filler group than in the collagen group (*p* < 0.05). These symptoms were mild and resolved within one week with conservative treatments such as oral analgesics and warm compresses (Table 5).

**Table 5:** The incidence of adverse local reactions at the injection sites within 30 days after injection

Local reaction	The mADM filler (n=86)	The collagen filler (n=89)	*p*-Value
Allergy	5 (5.8%)	3 (3.4%)	0.515
Redness	23 (26.7%)	12 (13.5%)	0.055
Bruising	10 (11.6%)	3 (3.4%)	0.116
Rash	0 (0.0%))	1 (1.1%)	1.000
Hardness/nodules	27 (31.4%)	21 (23.6%)	0.132
Itching	5 (5.8%)	7 (7.9%)	0.783
Hyperpigmentatio	5 (5.8%)	2 (2.3%)	0.301
Others	4 (4.7%)	0 (0.0%)	0.078
Pain	35 (40.7%)	9 (10.1%)	<0.001
Swelling	39 (45.4%)	12 (13.5%)	<0.001
Increased skin temperature	7 (8.1%)	1 (1.1%)	0.003

The incidence of swelling, pain, and skin temperature increase was significantly higher in the mADM filler than that in the collagen filler (*p *< 0.05).

## Discussion

Soft tissue fillers for NLF correction include both biodegradable materials such as collagen, hyaluronic acid (HA), poly-L-lactic acid (PLLA), polydioxanone (PDLLA), and polycaprolactone (PCL), as well as permanent synthetic polymer such as polymethylmethacrylate (PMMA). HA fillers are most widely used due to their safety, reversibility, and low incidence of adverse effects. However, they degrade over time, requiring repeated injections, which may lead to the delayed hypersensitivity reactions [9]. PLLA has the potential to cause visible nodules, especially with high doses or repeated injections, limiting its use in sensitive areas like the periocular and perioral regions [10]. PMMA provides permanent volume but requires prior allergy testing and may cause granulomas or scarring [11]. Collagen fillers are considered safe and biodegradable, suitable for correcting various wrinkles, including NLFs [12].

The mADM filler (Cymetra) was first introduced by LifeCell in the early 2000s and was used for treating soft tissue defects, complex diabetic sinus tract wounds [13] and vocal cord insufficiency [14]. As a bio-scaffold, the mADM filler works by an entirely different mechanism of action. Upon implantation and injection, it supports patient's own cells to recellularize and revascularize the matrix, and can remodel into the patient’s own site-appropriate tissues. Despite of the off-label use in various aesthetic and plastic procedures over 20 years [15–17], there is still lack prospective randomized controlled clinical study for the mADM facial filler. As far as we know, the present study is the first to compare an mADM facial filler with a regulatory agency-approved facial filler in a double-blind, multi-centre clinical trial.

In the present study, the mADM filler Regenfil achieved an efficacy of 88.4% and 70.9% in the WSRS improvement at three months and six months respectively, demonstrating the non-inferiority to the collagen filler Summax Collagen Inplant I-Plus. It should be noted that the efficacy was achieved even at lower filling volumes and fewer supplemental injections as compared to the collagen fillers (control device). But the use of the mADM filler for NLF correction registered significantly more incidences of mild adverse local reactions (e.g., pain, swelling, redness, hardening). These reactions were mild and self-limiting, with safety profiles similar to bio-stimulating fillers like PLLA [18, 19]. About half of the adverse local events in the mADM filler lasted less than a month, with 7 lasting over two months. The majority of these adverse local events (78%) resolved on their own without any treatment or lasting effects. The rest (22%) were treated with medication, resolving within 19 days without sequelae. The lower participant-reported GAIS improvement and participant satisfaction at 6-week timepoint could be related to these early adverse local events, since participants might take into consideration adverse local reactions in their evaluations. The GAIS improvement assessed by the injectors was significantly greater than the participant’s own evaluation. The long-term participant satisfaction rates and GAIS improvement rates showed no significant difference between the mADM filler and the collagen filler.

The causes of more pain, swelling, redness and hardening with the mADM filler remain to be investigated. The mADM filler is designed to serve as a bio-scaffold that reconstructs good spatial microenvironments for cell ingrowth and revascularization to regenerate the patient’s own site-appropriate tissues. To do so, it needs greater material rigidity and higher concentration to create the mechanical support. Therefore, the mADM filler consists of highly-dense irregular particles that are less compressible, as compared to soft collagen or hyaluronic acid gels. The mADM filler needs larger needles for injection (23G or 25G, instead of 27G for the collagen filler), also causing more pain. Another reason might be related to the role of immuno-modulation by mADM that induces new tissue formation. According to the animal studies conducted by the manufacturer (unpublished), the injected mADM materials undergoes a structural or state change during the first 4 weeks in vivo, which is associated to the infiltration of polymorphonuclear leukocytes, but with fewer lymphocytes and/or giant cells. This transformation results in the formation of the host’s new tissues in the injected area as the injected material degrades and remodels over a period of 8 to 12 weeks. No pre-testing of allergic reactions was performed prior to the mADM injection in this clinical trial. But the adverse local events are unlikely related to allergic and xenogeneic reactions to the decellularized and antigen-eliminated porcine mADM materials. Xenogeneic and/or allergic reactions to animal-derived medical products are often associated with the presence of residual alpha-Gal epitopes (alpha-Gal syndrome, AGS) and other bovine gelatin anaphylactic reactions (BG-woAG) [20]. The quantification of alpha-Gal epitopes with M86 antibody demonstrates a 99.8% reduction of alpha-Gal epitopes, and a residual number of alpha-Gal epitopes as low as 1.7 ± 0.3 × 10^12^ epitopes/mg dry mADM mass [21].While the mADM filler represents a new class of promising materials for truly regenerative facial rejuvenation, more studies are needed to understand the interplays between the host’s ‘sensing or recognition’ to the mADM material, mADM-induced inflammatory responses and tissue regeneration. Studies are also need to establish the appropriate method and guideline for mADM injection, such as better treatment course, filling volume, anatomic layers for injection, etc. A gradual facial rejuvenation through multiple applications with less filling volume each time may reduce the adverse local events.

It is worth noting that no complications related to vascular embolism occurred in the mADM filler, whereas vascular embolism is the primary complication that needs to be considered in traditional fat grafting and hyaluronic acid injection procedures.Vascular embolism is a serious complication that may occur during the injection of various fillers into the nasolabial folds, potentially leading to severe adverse effects such as local skin necrosis, vision impairment, or even vision loss. In this study, no clear symptoms of any vascular embolism were observed in both fillers. This observation may be related to the unique property of collagen molecules that enhance blood coagulation and form stable structures at the injection sites with its inability to swell and move.

The limitations of this study include the lack of data on the long-term efficacy of mADM beyond 6 months. Studies are on-going to collect the long-term efficacy data beyond six months. Different types of fillers have their own advantages and disadvantages, and comparative studies with other advanced fillers, such as mixed hyaluronic acid or composite fillers, would provide further evidence to optimize the treatment strategy for NLFs correction.

## Conclusion

The mADM filler (Regenfil) is safe and effective for treating moderate-to-severe NLFs, and can achieve comparable outcomes to the use of the collagen fillers even at less filling volume and fewer supplemental injections. However, more mild adverse local reactions are reported at the injection sites for the mADM filler than the collagen filler.

## Supplementary Information

Below is the link to the electronic supplementary material.

Supplementary file1 (DOCX 15 kb)

Supplementary file2 (DOCX 15 kb)

Supplementary file3 (DOCX 14 kb)

Supplementary file4 (DOCX 16 kb)
