# Supplementary material for: Safety and Efficacy of Micronized Acellular Dermal Matrix Injection for Correction of Moderate to Severe Nasolabial Folds: A Double-Blind, Multicenter, Randomized Controlled, Non-inferior Clinical Trial
Source: Aesthetic Plast Surg. 2025 Dec 11;50(10):3710–9. doi: 10.1007/s00266-025-05494-4 (PMC13219193; doi:10.1007/s00266-025-05494-4)
Supplement: Supplementary file 1 — Supplementary file1 (DOCX 15 kb) [file 266_2025_5494_MOESM1_ESM.docx]

**Supplementary Table 1. NLFs Assessment using the Wrinkle Severity Rating Scale (WSRS)**

| Grade | Severity Description |
| --- | --- |
| 1 | No visible NLF; only fine skin lines are present. |
| 2 | Shallow but visible NLF with slight indentation; fine facial lines; minor improvement expected. |
| 3 | Moderately deep NLF; clearly visible facial lines that disappear upon stretching; significant correction expected with injection. |
| 4 | Very deep and long NLF; prominent facial lines with less than 2 mm visible upon stretching; substantial correction expected with injection. |
| 5 | Extremely deep and long NLF causing severe facial distortion; 2–4 mm V-shaped folds visible upon stretching; satisfactory correction unlikely with injection. |
