# Supplementary material for: Safety and Efficacy of Micronized Acellular Dermal Matrix Injection for Correction of Moderate to Severe Nasolabial Folds: A Double-Blind, Multicenter, Randomized Controlled, Non-inferior Clinical Trial
Source: Aesthetic Plast Surg. 2025 Dec 11;50(10):3710–9. doi: 10.1007/s00266-025-05494-4 (PMC13219193; doi:10.1007/s00266-025-05494-4)
Supplement: Supplementary file 2 — Supplementary file2 (DOCX 15 kb) [file 266_2025_5494_MOESM2_ESM.docx]

**Supplementary Table 2. Global Aesthetic Improvement Scale (GAIS)**

| Grade | Evaluation | Description |
| --- | --- | --- |
| 1 | Very much improved | Optimal cosmetic result achieved for the participant. |
| 2 | Much improved | Significant improvement compared to baseline but not optimal for the participant. |
| 3 | Improved | Moderate improvement compared to baseline. |
| 4 | No change | Appearance is essentially the same as baseline. |
| 5 | Worse | Appearance has deteriorated compared to baseline. |
