# Supplementary material for: Safety and Efficacy of Micronized Acellular Dermal Matrix Injection for Correction of Moderate to Severe Nasolabial Folds: A Double-Blind, Multicenter, Randomized Controlled, Non-inferior Clinical Trial
Source: Aesthetic Plast Surg. 2025 Dec 11;50(10):3710–9. doi: 10.1007/s00266-025-05494-4 (PMC13219193; doi:10.1007/s00266-025-05494-4)
Supplement: Supplementary file 3 — Supplementary file3 (DOCX 14 kb) [file 266_2025_5494_MOESM3_ESM.docx]

**Supplementary Table 3. The Participant Satisfaction Survey Scale**

| Score | Satisfaction Level |
| --- | --- |
| 5 | Very satisfied |
| 4 | Satisfied |
| 3 | Neutral |
| 2 | Dissatisfied |
| 1 | Very dissatisfied |
