# Supplementary material for: Safety and Efficacy of Micronized Acellular Dermal Matrix Injection for Correction of Moderate to Severe Nasolabial Folds: A Double-Blind, Multicenter, Randomized Controlled, Non-inferior Clinical Trial
Source: Aesthetic Plast Surg. 2025 Dec 11;50(10):3710–9. doi: 10.1007/s00266-025-05494-4 (PMC13219193; doi:10.1007/s00266-025-05494-4)
Supplement: Supplementary file 4 — Supplementary file4 (DOCX 16 kb) [file 266_2025_5494_MOESM4_ESM.docx]

**Supplementary Table 4. The enrollment of participants in five hospitals**

| Research centres | Filler | Participants enrolled | Completed participants | Number of Drop-offs |
| --- | --- | --- | --- | --- |
| Peking University Third Hospital | mADM filler | 31 | 24 | 7 |
|  | Collagen filler | 30 | 26 | 4 |
| West China Hospital of Stomatology | mADM filler | 10 | 7 | 3 |
|  | Collagen filler | 10 | 8 | 2 |
| Beijing Friendship Hospital | mADM filler | 18 | 16 | 2 |
|  | Collagen filler | 18 | 15 | 3 |
| Zhujiang Hospital | mADM filler | 18 | 17 | 1 |
|  | Collagen filler | 19 | 17 | 2 |
| Peking Xiehe Hospital | mADM filler | 24 | 22 | 2 |
|  | Collagen filler | 24 | 23 | 1 |
| Total enrollment | mADM filler | 101 | 86 | 15 |
|  | Collagen filler | 101 | 89 | 12 |
